# [Retracted] Network pharmacology combined with experimental validation to investigate the effect of Rongjin Niantong Fang on chondrocyte apoptosis in knee osteoarthritis

**DOI:** 10.3892/mmr.2026.13957

**Published:** 2026-07-06

**Authors:** Jun Chen, Ting Zhang, Qingqing Luo, Ruyi Wang, Yuting Dai, Zhenyuan Chen, Chutian Zhang, Xuzheng Chen, Guangwen Wu

Mol Med Rep 29: 102, 2024; DOI: 10.3892/mmr.2024.13226

Following the publication of the above article, and a Corrigendum (doi: 10.3892/mmr.2025.13581) that was published to rectify errors associated with the published versions of Figs. 5, 8, 9 and 10, an interested reader contacted the Editorial Office to explain that they had identified additional issues in need of further investigation. Subsequently, the authors recognized that there were further problems associated with the manuscript requiring attention which arose while they were organizing materials related to this manuscript. To ensure the scientific integrity of the work, the authors have requested that this article now be retracted from the Journal. In view of these additional issues, the Editor of *Molecular Medicine Reports* has agreed to the authors’ request to retract this paper. Note that all the authors agree to this retraction, and the Editor and the authors apologize to the readership of the Journal for any inconvenience caused.

